# Codon usage clusters correlation: towards protein solubility prediction in heterologous expression systems in *E*. *coli*

**DOI:** 10.1038/s41598-018-29035-z

**Published:** 2018-07-13

**Authors:** Leonardo Pellizza, Clara Smal, Guido Rodrigo, Martín Arán

**Affiliations:** Laboratory of Nuclear Magnetic Resonance, Fundación Instituto Leloir, IIBBA-CONICET, Av. Patricias Argentinas 435, C1405BWE CABA, Argentina

## Abstract

Production of soluble recombinant proteins is crucial to the development of industry and basic research. However, the aggregation due to the incorrect folding of the nascent polypeptides is still a mayor bottleneck. Understanding the factors governing protein solubility is important to grasp the underlying mechanisms and improve the design of recombinant proteins. Here we show a quantitative study of the expression and solubility of a set of proteins from *Bizionia argentinensis*. Through the analysis of different features known to modulate protein production, we defined two parameters based on the %MinMax algorithm to compare codon usage clusters between the host and the target genes. We demonstrate that the absolute difference between all %MinMax frequencies of the host and the target gene is significantly negatively correlated with protein expression levels. But most importantly, a strong positive correlation between solubility and the degree of conservation of codons usage clusters is observed for two independent datasets. Moreover, we evince that this correlation is higher in codon usage clusters involved in less compact protein secondary structure regions. Our results provide important tools for protein design and support the notion that codon usage may dictate translation rate and modulate co-translational folding.

## Introduction

Heterologous protein expression has become one of the central fields in biochemistry, being both the scientific research and the biotechnology industry dependent on its success. Remarkable advances in genetic engineering have resulted in the development of bacterial expression systems capable of producing large amounts of proteins from cloned genes^1–3^. However, efficient expression of genes in heterologous systems is actually a major bottleneck. In fact, one of the main problems often occurring during recombinant protein production in bacteria is undoubtedly the incorrect folding of the nascent polypeptides, resulting in their aggregation and accumulation as insoluble inclusion bodies, making the purification process a laborious or impossible task. As a result, approximately 50% of proteins are not soluble when expressed in *E*. *coli*^4^.

During the last decade, computational methods have provided interesting tools to address heterologous protein production obstacles^5,6^. However, the predictive power of algorithms is still limited in many cases, and developers run into the challenge of extracting reliable datasets in terms of the nature of the data source^5^. Most prediction tools are based on datasets generated from *E*. *coli* proteome^7,8^. In consequence, they are not specifically developed to predict the solubility of proteins expressed heterologously in *E*. *coli*. In addition, even when datasets are gathered by integrating heterogeneous available databases, the experimental details are often not consistent and without proper annotations^9^. In this scenario, it becomes clear that improvements in protein expression and solubility prediction algorithms should be associated with the generation of more diverse datasets with standardized solubility measurements.

In this context, the bacterium *Bizionia argentinensis* represents an interesting source of new protein datasets to expand our current view on the factors that direct heterologous protein expression in *E*. *coli*. *B*. *argentinensis* is a psychrotolerant bacterium, defined as a mesophilic organism that can tolerate low temperatures, with optimal growth temperature between 22 and 25 °C^10^. In addition, this bacterium is phylogenetically distant from *E*. *coli*, since it is classified in a different phylum (Bacteroidetes). We here present a study of the heterologous expression in *E*. *coli* of a set of selected proteins form *B*. *argentinensis*. We show for the first time a quantitative study of the total expression and solubility of thirty proteins from a psychrotolerant organism. We found that 50% of the expressed proteins could be classified as soluble, being this value remarkably similar to that previously described for proteins of thermophilic and mesophilic organisms^11,12^. In addition, we evaluated the influence of different factors, known to modulate heterologous protein production, on the experimental expression and solubility of our dataset. A significant positive correlation was found between the Codon Adaptation Index (CAI) and the total expression of the selected targets (r = 0.464, p = 0.017). In search of other unknown features related to the experimental solubility, we relayed on the concept of “codon harmonization” to apply the %MinMax algorithm. We defined Δ%MinMax and %MinMax Correlation as two novel parameters to quantify the differences in %MinMax profiles between the host and the target genes. We found that the Δ%MinMax showed a significant negative correlation (r = −0.645, p = 7.10^−4^) with total expression levels. But most importantly, a strong positive correlation (r = 0.787, p < 1.10^−4^) between the solubility of the selected proteins and the %MinMax Correlation was observed. Further analysis on the predicted secondary structure of the selected ORFs showed that %MinMax Correlation in codon clusters specifically involved in coil and β-sheet structures displayed the highest correlation with solubility. The predictive capacity of these parameters in the expression and solubility of an independent dataset of mesophilic prokaryotic proteins was evaluated.

Our results provide novel tools to study the factors governing protein solubility and support the notion that codon usage may dictate translation rate and modulate co-translational folding. Moreover, we here evince that the conservation of codon usage clusters in less compact protein secondary structure regions (coils or β-sheets) is one of the most important factors that determine recombinant protein solubility.

## Results

### The solubility yields of *B*. *argentinensis* proteins produced in *E*. *coli* are comparable with those of thermophilic and mesophilic organisms

One of the most challenging steps in heterologous protein expression is predicting which protein or protein fragment will express in a soluble form and purify. Considerable achievements have been made by several structural genomics (SG) initiatives where target selection was mainly based on a standardized bioinformatics pipeline which eliminates proteins bearing trans-membrane segments, signal peptides and large disordered regions^13^. In this context, to subsequently compare our results on the expression and solubility of proteins of *B*. *argentinensis* with those of previous studies, we selected 30 open reading frames (ORFs) that met the following characteristics: (i) low sequence relatedness to proteins of known function, but presenting counterparts in the genomes of other organisms (so-called “conserved hypothetical proteins”), (ii) without homologous of known structure deposited in the Protein Data Bank (PDB) and (iii) predicted cytosolic or extracytoplasmic localization (Supplementary Table S1).

In order to evaluate the expression and solubility of recombinant proteins, the 30 selected ORFs from *B*. *argentinensis* were cloned and expressed in *E*. *coli* BL21 (DE3) cells at different induction temperatures, as detailed in Materials and Methods. After induction, soluble and insoluble protein fractions were prepared and visualized by SDS-PAGE. Bands with the expected molecular masses were clearly evident for all expressed ORFs (Supplementary Fig. S1).

**Figure 1:**
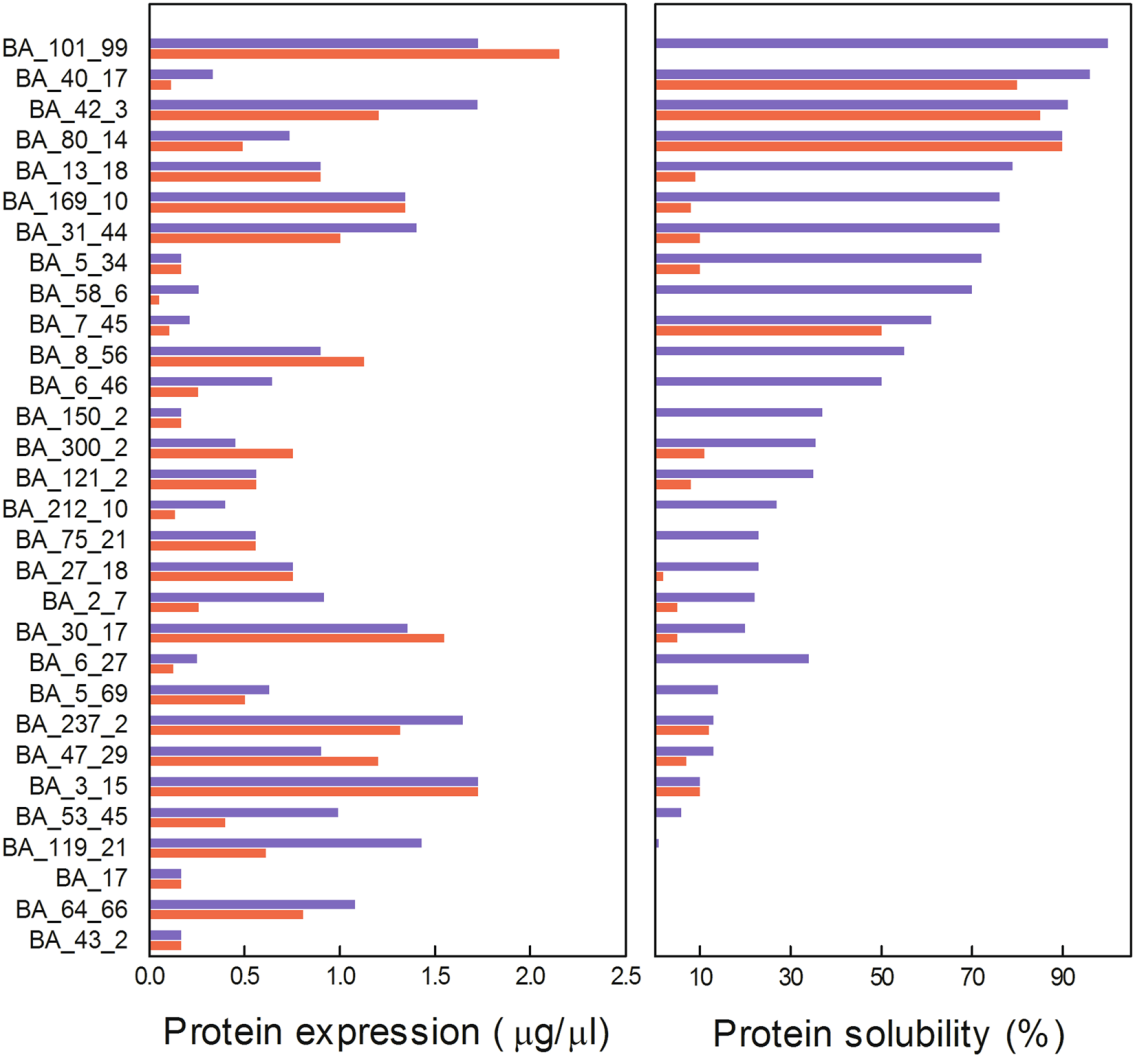
Quantitative analysis of the total expression and solubility of the selected targets. Total expression levels and percentages of solubility were estimated using densitometric analysis of the induced bands present in the pellet and supernatant of SDS-PAGE. Bars plot of total expression and solubility of the selected targets induced at 37 °C (red bars) or 20 °C (blue bars) are shown.

To further analyze the behavior of selected targets from a quantitative point of view, total expression levels and percentages of solubility were estimated using densitometric analysis of the induced bands present in the pellet and supernatant of SDS-PAGE (Fig. 1). Overall, we found that most selected targets increased their proportion in the soluble fraction when the induction temperature was set at 20 °C. In contrast, when induced at 37 °C recombinant proteins were mainly present in the insoluble extract (Fig. 1). Therefore, taking into account the solubility at 20 °C and establishing a 30% threshold value, as previously reported by Niwa *et al*.^14^, 15 of the 30 proteins were classified as soluble. Interestingly, the value of 50% of solubility was in line with those typically reported by structural genomics projects based on *E*. *coli* expression systems of mesophilic and thermophilic organisms^15^.

Contrary to the behavior observed for solubility, we were unable to find a clear relationship between total expression levels and the induction temperature (Fig. 1). The expression levels of half of the expressed proteins were increased at 20 °C, when compared with 37 °C. In contrast, 17% of the targets analyzed exhibited higher expression levels at 37 °C. The remaining 33% of ORFs showed no differences in their total expression levels, regardless of the induction temperature used.

Taken together, these results clearly indicated that total expression levels and solubility were no correlated in our dataset. Moreover, in contrast to total expression, protein solubility showed an evident dependence on the induction temperature. In addition, the loss of half of the targets highlighted the main obstacle in the expression of soluble heterologous proteins.

### Solubility prediction algorithms have a limited predictive power on *B*. *argentinensis* proteins

Several attempts have been made to predict the solubility of recombinant proteins based on sequence^7–9,16^. Most algorithms have shown some predictive success for *E*. *coli* proteins and use several features to predict protein solubility such as: type of amino acid, protein length, isoelectric point (pI) and hydropathy index, among many others^5^.

In this context, to evaluate the predictive capacity of available tools on the solubility of our experimental dataset, we selected four freely prediction online programs: Protein-Sol^7^, CCSOL^8^, SOLpro^9^ and Recombinant protein solubility prediction^17^. The performances of these tools were tested by measuring the Matthews Correlation Coefficient (MCC) and the prediction accuracy. Table 1 shows the values obtained using three different test datasets: (i) the reported original datasets^7–9,17^, (ii) a merged dataset from multiple sources previously described by Chang *et al*.^6^ and (iii) our dataset. Interestingly, the accuracy values obtained for our experimental dataset were between 50 and 57%, in contrast to those reported for each program, which were between 74 and 87%. Accordingly, the calculated MCC values were clearly lower than those previously reported (Table 1). However, the prediction accuracy and MCC values obtained with our independent dataset were strikingly similar to those reported by Chang *et al*.^6^.

**Table 1:** Predictive capacity of available tools on the solubility of different experimental data sets.

Tool	RPSP	Protein Sol	CCSOL	SOLpro
**Original references**				
PA (%)	87.0	82.8 (*)	76.1	74.2
MCC	n/a	n/a	0.519	0.487
**Chang** ***et al***.^6^				
PA (%)	51.45	n/a	54.20	59.9
MCC	0.029	n/a	0.084	0.202
**This paper**				
PA (%)	56,6	53.3	50	50
MCC	0.151	0.089	0	0

The performances of four freely prediction online programs: Protein-Sol^7^, CCSOL^8^, SOLpro^9^ and Recombinant protein solubility prediction (RPSP)^17^ were evaluated. Three different test datasets were used: (i) the reported original datasets^7–9,17^, (ii) a merged dataset from multiple sources previously described by Chang *et al*.^6^ and (iii) the dataset from this paper. PA: Prediction accuracy; MCC: Matthews Correlation Coefficient; n/a: not available; *PA using 58% solubility prediction threshold.

Collectively, the results obtained with these prediction programs, which are mainly based on physicochemical properties of amino acids, revealed a limited predictive power not only for our set of proteins from a psychrotolerant organism, but also for other independent dataset with most mesophilic and thermophilic organisms^6^.

### The Codon Adaptation Index, but not the mRNA stability, correlates with total expression levels of *B*. *argentinensis* proteins

Codon usage bias and mRNA structural stability have been identified as two of the most important factors that influence heterologous protein expression and solubility in *E*. *coli*^18,19^.

**Figure 2:**
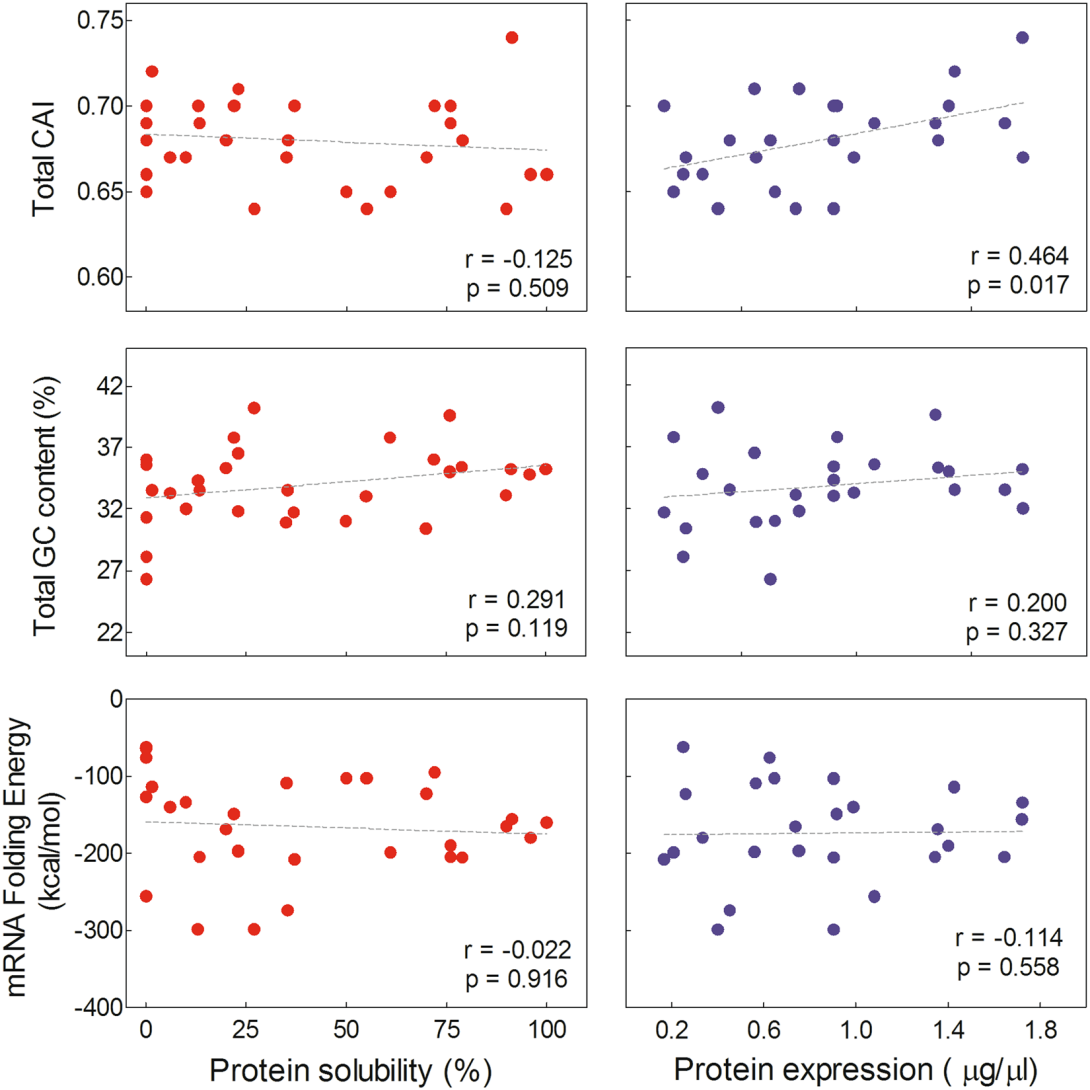
Analysis of primary determinants of gene expression. The total CAI, GC content and mRNA folding energy are plotted as a function of the experimental total expression (blue circles) and solubility (red circles). The linear regression (dashed line), the Pearson’s correlation coefficient and the p-value (two tailed) are shown.

Codon bias occurs from the different frequencies of synonymous codons in the coding DNA sequences that often mirrors the amount of the cognate tRNAs. Various estimators were developed in order to quantify the codon bias between a coding sequence and a set of reference sequences. One of the most widespread parameters is the CAI^20^. However, although high CAI has been associated to high expression levels^21,22^, some contradictory reports have been published^23^. In addition, several studies in heterologous protein expression systems using CAI on codon optimization of individual genes have not addressed protein solubility^24,25^. In this context, we calculated the CAI for all selected ORFs in order to analyze the influence of codon usage in protein expression and solubility of our dataset. We found a significant positive correlation between the CAI and the total expression levels (r = 0.464, p = 0.017) (Fig. 2). On the other hand, no significant correlation was observed between CAI and solubility (Fig. 2).

Among other factors proposed as primary determinants of gene expression, mRNA stability has been of particular interest^26,27^. Therefore, we estimated mRNA stability from both the predicted folding free energy of the mRNA and the GC content for the native coding sequences. We next analyzed the relationship between these two global parameters and the total expression level and solubility of our dataset. Notably, neither the GC content nor the mRNA folding energy were significantly correlated with total expression or solubility of the protein targets (Fig. 2).

### Two novel parameters derived from the %MinMax algorithm are strongly correlated with solubility and total expression levels of recombinant proteins

**Figure 3:**
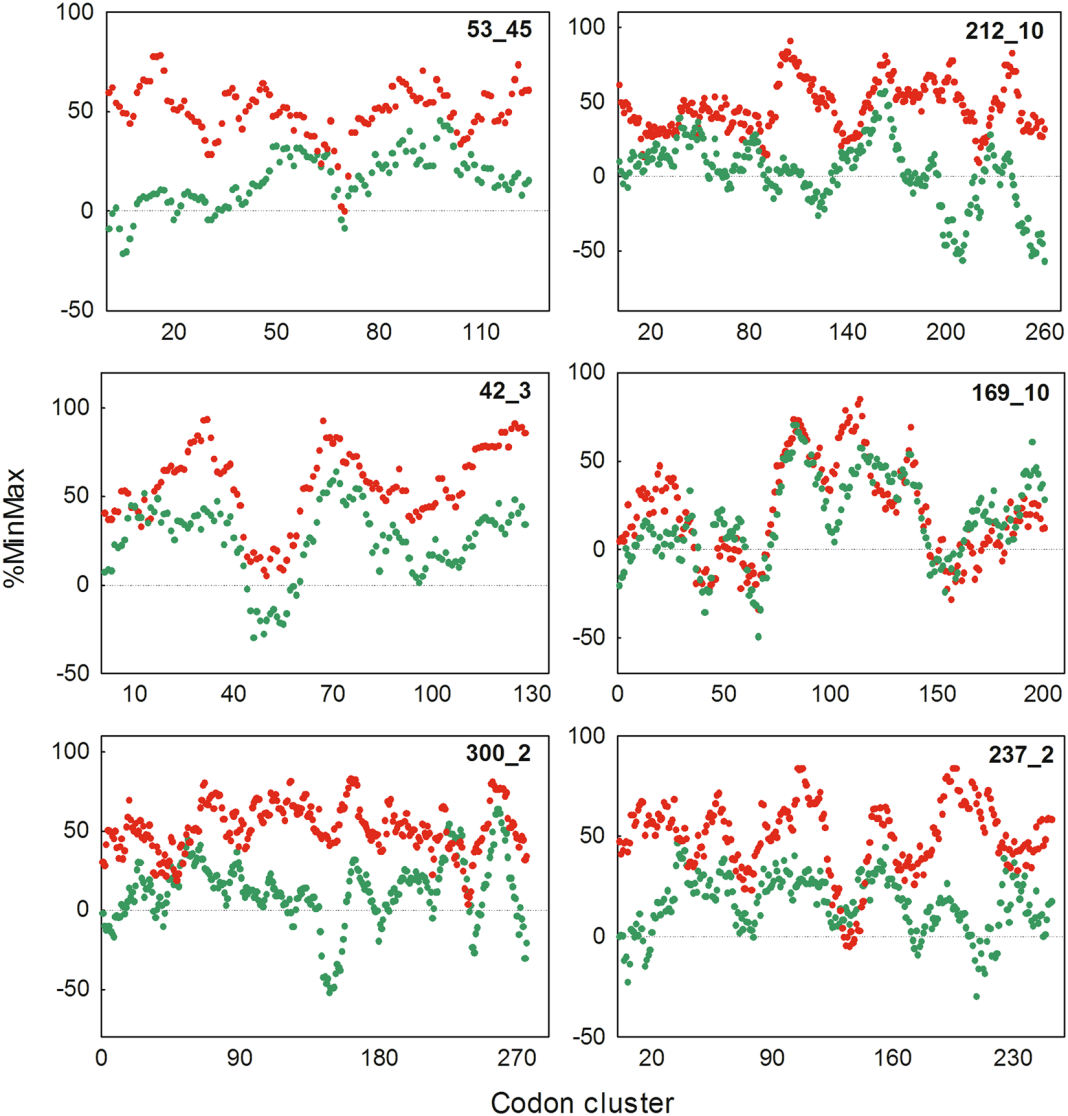
%MinMax profiles of protein targets. The %MinMaxBA (red circles) and the %MinMaxEC (green circles) for six representative ORFs are plotted and superimposed as a function of the codon cluster. %MinMaxBA and %MinMaxEC were calculated using *B. argentinensis* and *E. coli* codon usage frequency, respectively.

From the results described in the preceding sections, we were unable to find any significant correlation between the experimental solubility of the selected targets and the properties evaluated from their sequences. In search of other unknown features related to the experimental solubility, we relayed on the concept of “codon harmonization”. This strategy involves identifying significant patterns of synonymous codon usage in the host organism and replicating these patterns using the codon usage frequencies of the heterologous expression host^28^. It have been reported that soluble expression of the “harmonized” genes exceeded that of the native genes by 4- to 1,000-fold^29^. In this sense, the %MinMax algorithm serves as a useful tool in “codon harmonization” strategies, since it evaluates synonymous codon usage patterns for any coding sequence^28^. Therefore, we applied the %MinMax algorithm^30^ to selected ORFs in order to investigate the relationship between codon bias and the experimental solubility and expression levels. We calculated the %MinMax using *B*. *argentinensis* codon usage frequency (%MinMaxBA) or *E*. *coli* codon usage frequency (%MinMaxEC) for all selected ORFs, as described under Materials and Methods. In Fig. 3 six representative graphs are displayed, where the %MinMaxBA and %MinMaxEC for each ORF are superimposed and plotted as a function of the codon cluster. Notable, we found that the average %MinMaxBA was higher than the average %MinMaxEC for most ORFs analyzed. However, in some cases, such as for the ORF 169_10 (Fig. 3), both profiles were found to be remarkably similar to each other, not only considering the %MinMax average, but also the %MinMax for each particular cluster.

**Figure 4:**
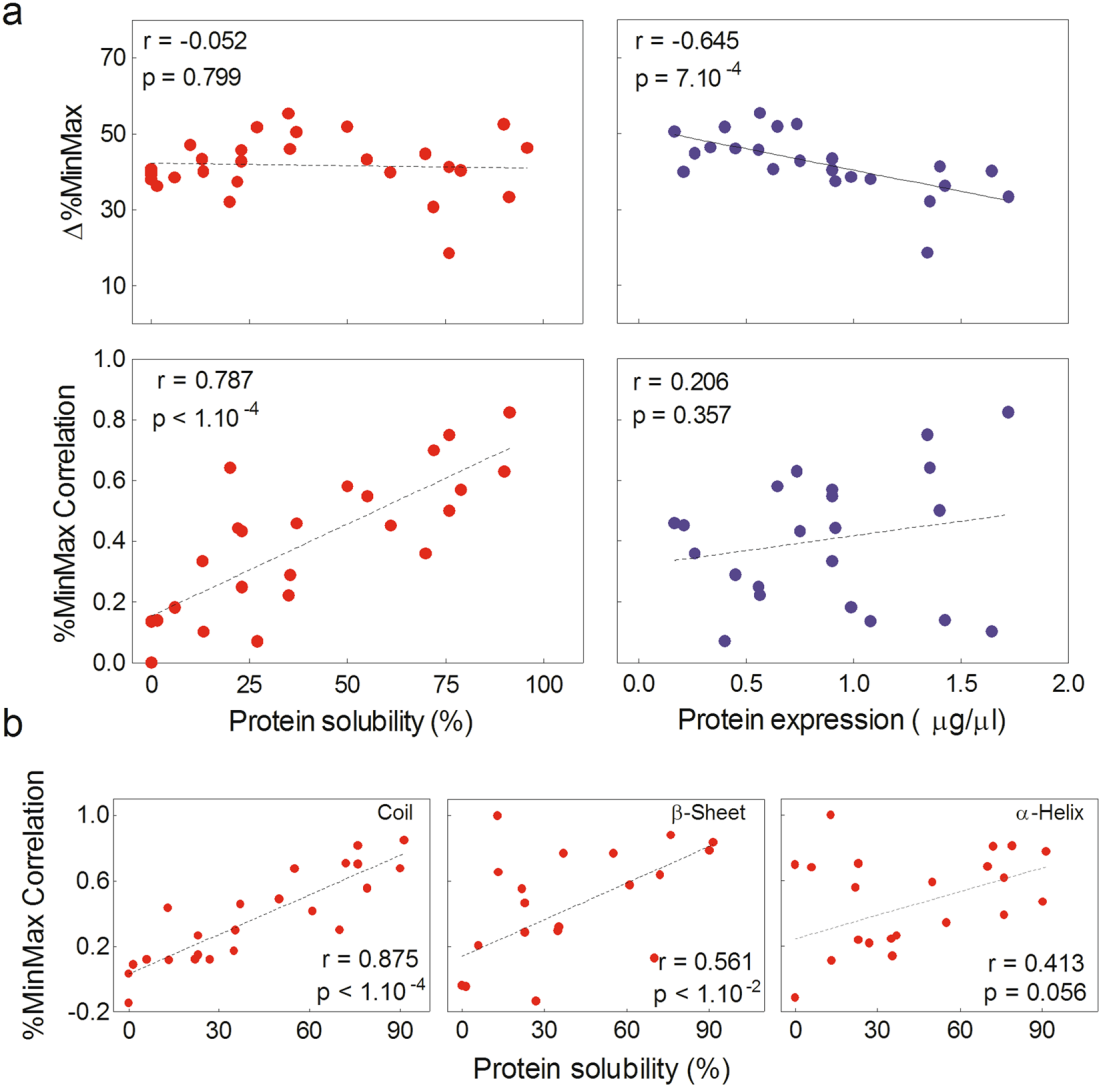
Analysis of %MinMax-derived parameters and their relationship with the solubility, total expression and predicted secondary structures elements of recombinant proteins. (**a**) The %MinMax Correlation and the Δ%MinMax calculated for each protein are plotted as a function of the experimental solubility (red circles) and total expression levels (blue circles). (**b**) The secondary structure content of all selected proteins was predicted using the JPred^33^. The %MinMax Correlation calculated for α-helices, β-sheets and coils are plotted as function of the experimental solubility. In A and B the linear regression (dashed line), the Pearson’s correlation coefficient and the p-value (two tailed) are shown.

We next evaluated whether the absolute difference between %MinMaxBA and %MinMaxEC (Δ%MinMax) for each ORF correlated with its solubility and total expression levels. Interestingly, we found that the Δ%MinMax showed a significant negative correlation with total expression levels (Fig. 4a). This result was in line with the previously observed for the CAI, as in both cases the smaller the differences between the codon frequencies of the host and the target gene, the higher the expression levels of the heterologous proteins. However, we were unable to find any significant correlation between the experimental solubility and the Δ%MinMax. At this point, from a detailed inspection of the %MinMax profiles, we noted that those ORFs that showed similar landscapes between %MinMaxBA and %MinMaxEC (i.e. in relative but not absolute terms) were expressed mostly soluble. The ORFs 169_10 and 42_3 were good examples (Fig. 3), being highly soluble at 20 °C and showing matching landscapes between the host and the target genes (Fig. 3). Consequently, in order to quantify these observations, we analyzed the correlation between %MinMaxBA and %MinMaxEC (%MinMax Correlation) for each ORF using the Pearson’s correlation coefficient as detailed in Materials and Methods. Surprisingly, a strong positive correlation was observed between the solubility of the selected proteins and the %MinMax Correlation (Fig. 4a). In other words, the greater the similarity between %MinMaxBA and %MinMaxEC landscapes (regardless of the magnitude of Δ%MinMax), the greater the proportion of the proteins found in the soluble fraction. In consequence, in contrast to the physicochemical properties of the polypeptide chain and other characteristics associated with mRNA stability analyzed, our results revealed that %MinMax Correlation was the only parameter that significantly correlated with the experimental solubility of our set of selected proteins.

### The correlation between protein solubility and %MinMax Correlation is higher in codon clusters associated to coil structures

Previously, the role of rare codons has been explored in relation to the structure of proteins. It was shown that optimal codons are preferentially used in gene regions that encode well-structured protein domains (mainly in α-helical regions) and more non-optimal codons are used in disordered/weakly structured regions (including coil, β-sheet and intrinsically disordered structures)^31,32^. In light of these results, we wondered if %MinMax Correlation and protein solubility could be related to the structure of the selected proteins. Therefore, we first predicted the secondary structures of all selected proteins using JPred^33^. This web server classifies each amino acid residue as belonging to α-helix, β-sheet or not α-helix or β-sheet secondary structures. In particular, we classified amino acids within this latter group as coil, also including residues in intrinsically disordered regions. We next differentiated the %MinMax Correlation in each protein accordingly to its secondary structure prediction. Finally, the Pearson’s correlation between %MinMax Correlation for the three classes of secondary structures and the experimental solubility was evaluated. Surprisingly, a strong positive correlation (r = 0.875, p < 1.10^−4^) was found between solubility and %MinMax Correlation in coil regions (Fig. 4b). In addition, a modest but significant positive correlation (r = 0.561, p < 1.10^−2^) was also observed between solubility and %MinMax Correlation in β-sheet structures (Fig. 4b). However, no significant correlation was detected between %MinMax Correlation and solubility in α-helical regions. These results were not biased due to the relative content of secondary structure in the target proteins, since the total percentage for coils, α-helices and β-sheets were 45%, 38% and 17%, respectively. Therefore, these data suggested that the greater the similarity between %MinMaxBA and %MinMaxEC landscapes in codon clusters specifically involved in coil (and to a lesser extent in β-sheet) structures, the greater the proportion of the proteins found in the soluble fraction.

### The use of Δ%MinMax and %MinMax Correlation in the study of an independent dataset of mesophilic prokaryotic proteins supports the results obtained for *B*. *argentinensis* proteins

On virtue of the results detailed in the previous section, we wondered if the %MinMax Correlation and Δ%MinMax could be applied to predict the solubility and total expression levels of other independent datasets. In this regard, we performed a search of heterologous proteins produced in *E*. *coli* with experimental expression and solubility values reported. After an exhaustive exploration, we only found a few public databases providing experimental information on the solubility of recombinant proteins. Among them, the SPINE system for structural proteomics, is a complete database that offers experimental information about expression systems, purification conditions and analytical measure of the solubility (http://spine.nesg.org)^34^. Based on the SPINE database, we generated a new dataset of 30 mesophilic prokaryotic proteins with reported expression values and known structure (see Supplementary Table S2). We next calculated the %MinMax Correlation and Δ%MinMax for all selected ORFs and analyzed their relationship with solubility and expression levels. Interestingly, in line with our previous results, the Δ%MinMax showed a significant negative correlation with total expression levels (r = −0.511, p = 7.10^−3^) (Supplementary Fig. S2). But most importantly, a strong positive correlation between the solubility of this independent dataset and the %MinMax Correlation was observed (r = 0.642, p < 1.10^−4^) (Supplementary Fig. S2).

Finally, we analyzed the relationship between %MinMax Correlation and protein solubility as a function of the secondary structure of the selected proteins. Surprisingly, the experimental solubility and the %MinMax Correlation were positively correlated for the three classes of secondary structures (Supplementary Fig. S1). In this case, differently from what was observed for the *Bizionia argentinensis* dataset, %MinMax Correlation in β-sheet regions showed the highest correlation with solubility (r = 0.696, p < 1.10^−4^), followed by α-helix (r = 0.567, p < 2.10^−3^) and coil regions (r = 0.472, p < 8.10^−3^).

Collectively, these results support the idea that there is a positive correlation between the solubility of recombinant proteins produced in *E*. *coli* and the %MinMax Correlation parameter. Moreover, they show that this correlation is higher if certain regions of secondary structure are taken into account. In addition, they reinforce the notion that there is a negative correlation between protein expression levels and Δ%MinMax.

## Discussion

Numerous methods have been proposed to predict the solubility of recombinant proteins overexpressed in *E*. *coli* merely from amino acid sequences. Although some of these models have acceptable prediction performances^5,6^, we found a poor predictive power of the four available algorithms tested. Moreover, our results were consistent with a previous analysis performed with an independent dataset^6^. Bearing in mind these observations and considering the influence of the codon bias on the solubility of our dataset (see below), we hypothesized that the lack of predictive capacity of these programs may be based on two main arguments. First, most algorithms are principally developed on information from *E*. *coli* proteins, whose codon frequency is already optimized to be produced with the same machinery^6^. Therefore, the weight of the physicochemical properties in the prediction of solubility could be relatively overestimated. And second, the solubility information provided by the majority of databases it is not generated using a single reliable protocol and different criteria are taken by developers to classify proteins into soluble and insoluble categories. Consequently, not only misclassification of proteins in this binary system (soluble-insoluble) could arise, but also valuable information concerning diverse “degrees of solubility” for each molecule could be lost.

In this work, we define Δ%MinMax and %MinMax Correlation as two novel parameters to quantify the differences in %MinMax profiles between the host and the target genes. To our knowledge, this is the first time that these parameters are employed in the study of protein expression in a heterologous system. Our results suggest that total expression and solubility of prokaryotic proteins produced in *E*. *coli* can be studied independently by specific parameters.

On the one hand, the absolute difference of the mathematical average of all codon usage frequencies between the host and the target gene seems to be a relevant parameter to predict total expression levels. This assumption is in agreement with our results with total CAI and supports the notion that the more codons that a gene contains that are rarely used in the expression host, the less likely is that the heterologous protein will be expressed at reasonable levels. There are several studies on gene expression of codon-optimized sequences, including mammalian proteins, which support this idea^21^.

On the other hand, the solubility appears to be associated to the magnitude of the correlation between the %MinMax profiles of the host and the target gene. On this basis, it can be predicted that an overall increase of high-frequency-usage codons in the target gene, in favor of enhanced total expression, may be detrimental to the solubility of the encoded protein, since such increment will be not necessarily associated with an improvement in %MinMax Correlation. In line with this assumption, the aggregation of several recombinant proteins has been effectively observed when “one amino acid-one codon” strategy was applied in order to optimize protein expression^19,35^. In contrast, a strategy aimed at increasing high-frequency-usage codons in the target gene while maximizing %MinMax Correlation would generate higher levels of total expression without affecting, or even improving, the amount of the recombinant protein in the soluble fraction. In this regard, in the codon harmonization strategy^29^ any change in the codon usage frequencies of the target gene necessarily mirrors the wild type %MinMax profile and, consequently, it would have less negative effects on the solubility of the recombinant proteins. This strategy has been successfully applied to express several proteins in *E*. *coli*, including protein based vaccines^36–38^. Further experiments will be needed, however, in order to determine the effect of increasing high-frequency-usage codons in the target gene, while maximizing %MinMax Correlation, but above the harmonized %MinMax profile.

Generally, rare codons are associated with slower rates for protein synthesis, and are typically considered deleterious for efficient protein production^20^. The predominant view holds that selection favors common codons, but a low level of rare codons is incorporated due to random mutational drift and weak selection^39^. However, recently reports suggest that clusters of synonymous rare codons are non-randomly widespread in the coding sequences of most prokaryotic and eukaryotic species^30,40^ and are conserved within homologous genes^41^. Altering synonymous codon usage has been shown to influence the expression level^42^, solubility^43^, co-translational modifications^44^ and targeting of encoded proteins^45^. Further, codon usage can also indirectly impact the translational efficiency of coding sequences by affecting mRNA structure at 5′ ends of transcripts^23,46^. In this scenario, our results clearly reinforce the view that synonymous codons clusters distribution in coding sequences is subjected to evolutionary pressures. But most importantly, here we show that, alternatively to the relative frequency of the codons (i.e. if they are rare or highly frequent) at point positions in the coding sequence, the conservation of the entire codon cluster profile seems to be crucial for the solubility of recombinant proteins.

Protein synthesis is coordinated by maintaining the nascent polypeptide in a folding-competent conformation both by direct ribosome effects^47^ and the translation rate as dictated by codon usage^48^. In general, reducing translation rate will increase the time available for N-terminal portions of a protein to fold to a stable structure prior to the appearance of more C-terminal regions^22,28,38^. Changes in codon usage frequency in a heterologous expression host can lead to alterations in local protein synthesis rates^49^. From this perspective, our results are congruent with the notion that the conservation of %MinMax profiles between the host and the target gene may enhance the chances of achieving native local protein synthesis rates, thus preventing the appearance of unstable folding intermediates that could lead to inclusion bodies formation. In consonance with this idea, it has been shown that the protein translation rate and silent codon substitution can affect protein folding of expressed heterologous proteins^48,50^. In addition, a correlation between translationally optimal codons and structurally sensitive^51^ and aggregation-prone sites^52^ in proteins has been described. However, more studies are needed in order to unveil the specific forces that determine the rate of translation of each codon and its impact in co-translational folding *in vivo*.

Computational analysis of the available *E*. *coli* genome and protein structure databases identified that high-frequency-usage codons are mainly associated with structural elements such as α–helices, whereas clusters of lower frequency usage codons are more likely to be associated with β-sheets, coils, and disordered regions^31,32^. In this context, we here show that coils regions (and to a lesser extent β-sheets) of the most soluble proteins in our dataset display the highest %MinMax Correlation. Furthermore, in an independent dataset (composed of mesophilic organisms) β-sheet regions of the most soluble proteins exhibit the highest %MinMax Correlation. Therefore, our results indicate that the degree of conservation of wild type %MinMax profiles in less compact secondary structure regions (coils or β-sheets) is an important factor that could determine the solubility of recombinant proteins.

Studies of the prokaryotic ribosomal tunnel during protein synthesis support its role as an active modulator of nascent peptide secondary structure formation^53^. A range of structural and biophysical studies have indicated that certain nascent chains can form secondary-structure and even simple tertiary-structure motifs within the ribosome exit tunnel: the dimensions of the exit tunnel permit the formation of α-helices within the central and lower tunnel^53^. In this regard, our results are consistent with the idea that the elongation rates of α-helices regions are less influential in the general pathway that leads to a native co-translational folding. On the other hand, our data support the notion that elongation rates of secondary structures elements that are dependent on other regions of the nascent chain to stabilize (e.g. coils and β-sheets), need to follow an exquisite folding kinetics to explore the energy landscape to reach a native co-translational folding. Moreover, the relationship between %MinMax profiles in coil regions and solubility of *B*. *argentinensis* proteins may reflect the particular characteristics of these structures in cold-adapted organisms. Since coils tend to be more flexible in psychrophiles than in mesophilic and thermophilic counterparts^54^ and the flexibility of these structural elements is commonly involved in the catalytic cycle of psychrophilic enzymes^55^.

Understanding the factors governing protein solubility is important to grasp the underlying mechanisms and improve the efficiency of designing soluble proteins. Moreover, they may provide insight into protein aggregation and misfolding related diseases. Sequence-based methods can be considered as valuable tools to predict recombinant protein overexpression results before performing real laboratory experiments, thus saving time, labor and cost. Generating more accurate datasets, working on organisms other than *E*. *coli* and discovering novel influential features, are some considerations for future directions in the protein solubility prediction field.

## Materials and Methods

### Target selection

In a first step, we selected those ORFs from *B*. *argentinensis* genome^56^ that belonged to a Pfam family of unknown function. For this purpose, the HMMER program^57^, that implements hidden Markov models and compares them with the sequences provided, was used. As a result, 979 ORFs encoding proteins of low sequence identity with proteins of known function were selected. Subsequently, from this group, we selected those ORFs without homologous of known structure deposited in the PDB. In this sense, we performed a search with the BLASTp program (https://blast.ncbi.nlm.nih.gov) against the PDB and discarded the ORFs with significant hits (E-value ≤ 0.001). Next, we analyzed the existence of orthologous sequences of selected ORFs in KEGG GENES (http://www.genome.jp/kegg/genes.html). To this end, the Bidirectional Best Hits (BBH) method was used^58^, and those sequences presenting hits with E-values ≤ 0.00001 were selected. We next discarded the ORFs with predicted signal peptides and membrane localization, using the SignalP^59^ and PSORTdb programs, respectively. Finally the ORFs were filtered by size between 80 and 500 amino acids. As a result, 30 of the 3195 ORFs identified in the genome of *B*. *argentinensis* were selected (Supplementary Table S1).

### Cloning

All target genes were amplified by PCR using *B*. *argentinensis* genomic DNA as template and the primers listed in Supplementary Table S3. The PCR products were purified and subsequently amplified in a second PCR with the forward primer 5′-GGGACAAGTTTGTACAAAAAAGCAGGCTCGGAGAACCTGTACTTTCAG-3′ and the reverse primer 5′-GGGGACCACTTTGTACAAGAAAGCTGGGTTA-3′. Next, the final PCR products were recombined using the Gateway® BP Clonase® II enzyme mix into the pDONR-201 vector (Invitrogen). The plasmid DNA from positive clones were purified, confirmed by DNA sequencing and recombined into the pDest-527 expression vector using the Gateway® LR Clonase® II enzyme mix. pDest-527 was a gift from Dominic Esposito (Addgene plasmid #11518). This protein expression system allows the production of recombinant proteins in *E*. *coli* as fusions with a His6 tag at their N-terminal. In addition, the tobacco etch virus (TEV) protease digestion site was introduced in order to subsequently remove the N-terminal fusion tag.

### Protein expression and solubility screening

Small-scale expression assays were conducted in order to determine the solubility and total level of recombinant proteins. Chemically competent BL21 (λDE3) *E*. *coli* cells were transformed with 5 ng of each expression plasmid and grown overnight on agar plates with ampicillin (100 μg ml^−1^). 10 ml cultures in LB medium with ampicillin were started from isolated colonies and grown overnight at 37 °C. The day after, 10 ml of fresh LB medium were inoculated at a final OD_600nm_ of 0.05 with the starter cultures. The cells were grown at 37 °C for 2–3 h up to OD_600nm_ ≈ 0.5. Then, heterologous protein expression was induced by addition of 0.5 mM IPTG. After 4 h (37 °C) or 16 h (20 °C) of continuous growth, the final OD_600nm_ was recorded. Cells were centrifuged (5000 g, 10 min) and the pellet resuspended in 1 ml lysis buffer (20 mM Tris-HCl, 0.5 M NaCl, 40 μg ml^−1^ phenylmethylsulphonyl fluoride, pH 7.5) per unit of OD_600nm_. Cell suspensions were disrupted by sonication for 5 min on ice until complete cell lysis was achieved. After centrifugation (10000 g, 30 min, 4 °C), the supernatants were separated and analyzed for protein concentration by a standard procedure^60^. Supernatant samples containing 20 μg of total protein were analyzed by SDS-PAGE. Pellets were resuspended in the same amount of lysis buffer as the supernatants and equivalent volumes loaded onto the electrophoresis gels. In order to estimate protein molecular weight, the BenchMarkTM Protein Ladder (Life Technologies) was used.

### Expression levels and solubility quantification

The quantification of SDS-PAGE bands corresponding to recombinant proteins was carried out with the ImageJ program^61^. Gel bands were selected and the average grey value (M) and total area (A) were measure for each expressed protein. Protein intensity (I) in each band was calculated by multiplying M by A. The solubility of each ORF at a given temperature was calculated as follows:

1 $$\frac{Isf}{(Isf+Iif)}$$

where *Isf* is the intensity of the protein in the soluble fraction and *Iif*, the intensity of the protein in the insoluble fraction.

The total expression was estimated using the BenchMarkTM Protein Ladder (Life Technologies). In each case, we compare de intensity of one selected band of the marker (according to the size of the target protein) with the intensity of the band corresponding to the protein of interest.

### Solubility prediction tools

All prediction tools used provide open accessibility. The URL addresses to access Protein-Sol^7^, CCSOL^8^, SOLpro^9^ and Recombinant Protein Solubility Prediction^17^ were https://protein-sol.manchester.ac.uk/, http://tartaglialab.crg.cat/ccsol.php, http://scratch.proteomics.ics.uci.edu and http://www.biotech.ou.edu, respectively. The performance of each tool was assessed by the Prediction Accuracy and the Matthews Correlation Coefficient (MCC) using the following equations,

2 $$Prediction\,Accuracy=\frac{TN+TP}{TN+FP+FN+TP}$$

3 $$MCC=\frac{TP\times TN-FP\times FN}{\sqrt{(TP+FP)(TP+FN)(TN+FP)(TN+FN)}}$$

where TP is the number of true positives, TN the number of true negatives, FP the number of false positives and FN the number of false negatives.

The tools were evaluated by setting the threshold value for classification of soluble class at 30% for our experimental data solubility, as previously reported by Niwa *et al*.^14^ and 50% for the prediction tools.

### Codon adaptation index (CAI)

The CAI for each ORF was calculated using the GenScript Rare Codon Analysis Tool (available at https://www.genscript.com/tools/rare-codon-analysis). The CAI was estimated using the equation given by Sharp and Li^20^ as follows:

4 $$CAI=exp\frac{1}{L}\sum _{k=1}^{L}ln{w}_{c(k)}$$

where *L* is the number of codons in the gene and *w*_*c*(*k*)_ is the relative adaptiveness value (ω) for the k^th^ codon in the gene. CAI is usually used to measure the ω of the codon usage of a gene towards the codon usage of highly expressed genes. For this analysis, the coding sequence of each ORF, excluding the additional 5′-end encoding a His6 tag and a TEV protease digestion site, was used.

### Analysis of the mRNA folding energy

The minimum free energy of mRNA secondary structure was predicted with the NUPACK web application^62^. For this analysis, the coding sequence of each ORF, excluding the additional 5′-end encoding a His6 tag and a TEV protease digestion site, was used. All predictions were performed at the selected expression temperature of 20 °C.

### %MinMax calculation

%MinMax was calculated using the Rare Codon Calculator (http://www.codons.org/)^30^. Absolute codon frequencies were tabulated using codon usage data for each organism. For all organisms, with the exception of *B*. *argentinensis*, codon usage data were extracted from KazUSA (http://www.kazusa.or.jp/codon/)^63^. In the case of *B*. *argentinensis* the codon usage data were generated with the Countcodon program (http://www.kazusa.or.jp/codon/countcodon.html) from 1000 ORFs. Δ%MinMax was calculated for each ORF as follows:

5 $${\rm{\Delta }}{\rm{ \% }}MinMax=\frac{\sum _{i=1}^{n}|{x}_{i}-{y}_{i}|}{n}$$

where n corresponded to the total number of windows for each ORF (i.e. the total number of aminoacids minus 18), *x*_*i*_ was the %MinMax obtained with *B*. *argentinensis* codon usage frequency (%MinMaxBA) and *y*_*i*_ was the %MinMax obtained with *E*. *coli* codon usage frequency (%MinMaxEC) for the i^th^ window in each ORF. The Δ%MinMax for other organisms was calculated as mentioned above, but using the %MinMax obtained with the codon usage frequency according to each organism as x.

The secondary structure prediction for each protein was obtained from the sequence using Jpred4 (http://www.compbio.dundee.ac.uk/jpred/).

### Correlation analysis

The correlation between the different features and the experimental expression and solubility values was evaluated accordingly to the Pearson’s correlation method. Given two variables x and y, the Pearson’s correlation coefficient r can be calculated as follows:

6 $$r=\frac{{\sum }_{i=1}^{n}\,({x}_{i}-\bar{x})({y}_{i}-\bar{y})\,}{\sqrt{{\sum }_{i=1}^{n}\,{({x}_{i}-\bar{x})}^{2}}\sqrt{{\sum }_{i=1}^{n}\,{({y}_{i}-\bar{y})}^{2}}}$$

where n is the sample size, *x*_*i*_ and *y*_*i*_ are the independent variables, and $\bar{{\rm{x}}}$ and $\bar{{\rm{y}}}$ are the mean values.

In order to calculate the %MinMax Correlation for each ORF, we applied the Pearson’s correlation equation using the %MinMaxBA as x and the %MinMaxEC as y. The sample size n corresponded in this case to the total number of windows for each ORF (i.e. the total number of aminoacids minus 18). The %MinMax Correlation for other organisms was calculated as mentioned above, but using the %MinMax obtained with the codon usage frequency according to each organism as x.

The ORFs that presented values of Δ%MinMax and %MinMax Correlation greater than 3 times the 95% confidence interval of the linear regression fit were excluded from the analysis. When %MinMax Correlation was analyzed as a function of the predicted secondary structure, only those ORFs containing more than 5% of the evaluated secondary structure were included.

### Target generation from the SPINE dataset

In order to generate an independent dataset, 30 ORFs from different prokaryotic mesophilic organisms were selected using the SPINE server (http://spine.nesg.org)^34^. The targets were filtered by expression system, including the ORFs reported to be cloned in the wild type form into the expression vector pET-21 and expressed in *E*. *coli* BL21 (DE3) at 37 °C.

The values of expression and solubility in the SPINE database are reported as discrete values from 0 (no expression or insoluble protein) to 5 (high expression or soluble protein)^64^. Consequently, for targets with more than one expression or solubility value reported, the final values of solubility and total expression were averaged.

### Data availability

The datasets generated during the current study are available from the corresponding author on reasonable request.

## Electronic supplementary material

Supplementary Information
