# Supplementary material for: Codon usage clusters correlation: towards protein solubility prediction in heterologous expression systems in E. coli
Source: Sci Rep. 2018 Jul 13;8:10618. doi: 10.1038/s41598-018-29035-z (PMC6045634; doi:10.1038/s41598-018-29035-z)
Supplement: Supplementary file 1 — Supplementary Information [file 41598_2018_29035_MOESM1_ESM.pdf]

SUPPLEMENTARY INFORMATION

Codon usage clusters correlation: towards protein solubility prediction in heterologous expression systems in *E. coli*

Leonardo Pellizza, Clara Smal, Guido Rodrigo, Martín Arán

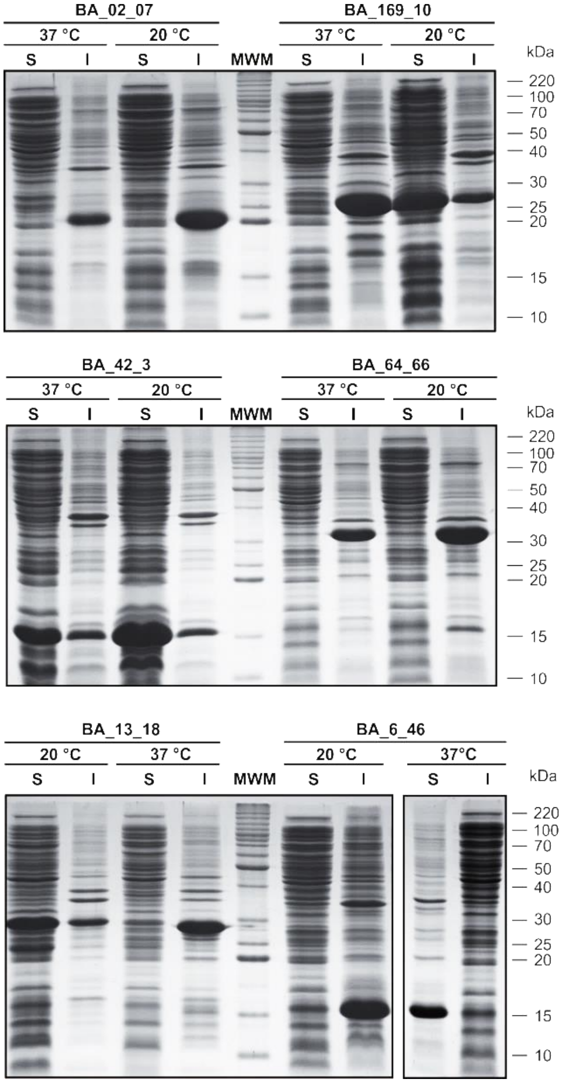

Figure S1: Expression distribution of protein targets between the soluble and insoluble fractions. Recombinant *B. argentinensis* proteins were expressed in *E. coli* (BL21) cells at different induction temperatures, as indicated. Cell suspensions were disrupted by sonication and the soluble (S) and insoluble (I) fractions were separated by centrifugation. Protein samples were subjected to SDS-PAGE and visualized by Coomassie blue staining. The BenchMarkTM Protein Ladder (Life Technologies) was used as reference (MWM). The results for six representative proteins are shown. The gel slice showing the expression distribution of the protein BA_6_46 induced at 37 °C was cropped from gel d of Figure S4. All gels images were taken from full-length gels of Figure S4.

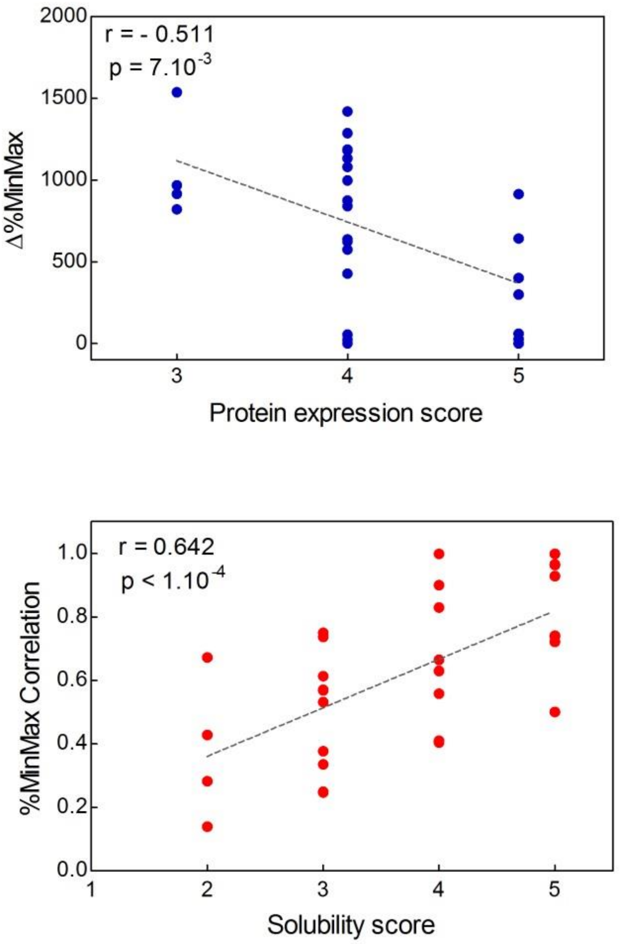

Figure S2: Evaluation of %MinMax Correlation and ∆%MinMax using an independent dataset. The independent dataset was randomly selected and filtered from the SPINE database. The %MinMax Correlation and ∆%MinMax calculated for 30 selected mesophilic prokaryotic proteins are plotted against the reported solubility (red circles) and total expression levels (blue circles). The Pearson´s correlation coefficient and the p-value (two tailed) are shown.

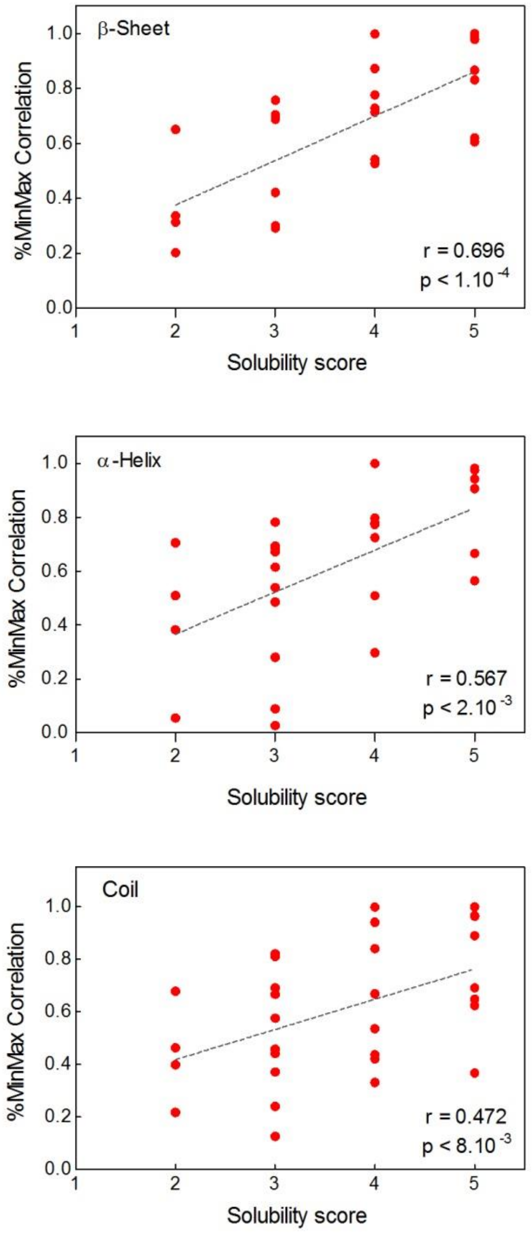

Figure S3: Relationship between the reported solubility and the %MinMax Correlation in predicted secondary structures elements of an independent dataset. The secondary structure content of all selected proteins was predicted using the JPred. The %MinMax Correlation calculated for α-helices, β-sheets and coils of 30 selected mesophilic prokaryotic proteins are plotted as function of the solubility score reported at the SPINE database**.** The Pearson´s correlation coefficient and the p-value (two tailed) are shown.

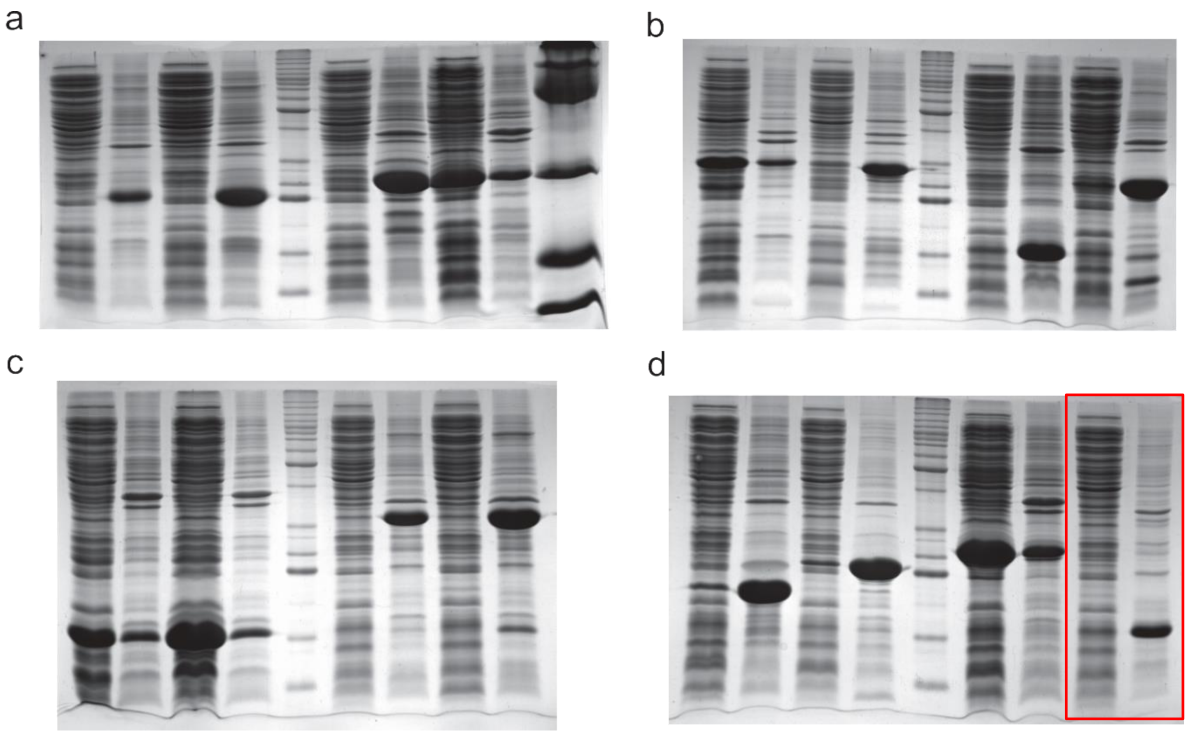

Figure S4: Full-length SDS-PAGE gels of the expression distribution of protein targets between the soluble and insoluble fractions used in Figure S1. Full-length gels for BA_02_07 and BA_169_10 (a), BA_13_18 and BA_6_46 (b), BA_42_3 and BA_64_66 (c) and BA_6_46 induced at 37 °C (d, highlighted in red box) are displayed.

Table S1: List of targets selected from *Bizionia argentinensis* genome

ORF ID	RefSeq ID	MW (kDa)	pI
BA_3_15	WP_008636253.1	18,2	4,78
BA_5_34	WP_040288292.1	12,7	4,08
BA_6_46	WP_008638276.1	14,1	4,93
BA_8_56	WP_008638493.1	14,1	4,76
BA_13_18	WP_008636370.1	25,8	7,26
BA_27_18	WP_008636361.1	26,9	8,43
BA_30_17	WP_008636271.1	20,8	6,53
BA_31_44	WP_008637983.1	23,5	5,38
BA_47_29	WP_008637057.1	35,7	5,04
BA_53_45	WP_008638147.1	16,1	4,75
BA_212_10	WP_008635676.1	29,7	5,96
BA_300_2	WP_008634504.1	32,9	5,79
BA_2_7	WP_008635241.1	17,6	5,00
BA_5_69	WP_008638919.1	12,4	5,16
BA_6_27	WP_008636968.1	9,9	8,69
BA_7_45	WP_008638142.1	23,2	5,21
BA_40_17	WP_008636292.1	23,1	8,83
BA_43_2	WP_008634534.1	15,8	5,03
BA_58_6	WP_008635118.1	18,3	5,22
BA_64_66	WP_008638663.1	29,7	5,40
BA_80_14	WP_008635870.1	20,8	7,88
BA_17	WP_008638724.1	8,6	5,73
BA_119_21	WP_008636681.1	13,8	4,93
BA_121_2	WP_008634530.1	13,9	8,98
BA_42_3	WP_008634846.1	16,5	4,72
BA_101_99	WP_008639670.1	19,2	5,73
BA_75_21	WP_008636597.1	21,2	4,64
BA_150_2	WP_008634508.1	26,4	9,03
BA_169_10	WP_008635470.1	25,7	6,78
BA_237_2	WP_008634494.1	31,8	7,66

MW: Molecular weight; pI: Isoelectric point. ORF ID: Code used to name *B. argentinensis* genes according to the following format: *BA_contig number_ORF number*.

Table S2: List of targets selected from SPINE database

SPINE ID	RefSeq ID
SR384	NP_389671.1
SR525	NP_390222.1
SR482	NP_391415.1
SR399	NP_389912.1
SR220	NP_390711.1
SR181	NP_389705.1
PAR319a	NP_250284.1
PAR365C	NP_251257.1
BFR322	YP_210255.1
BFR250	YP_211309.1
BFR257C	YP_211906.1
STR106	NP_459361.1
STR109	_
STR65	NP_459322.1
STR221	NP_462494.1
SFR7	NP_836168.1
SFR170	NP_836685.1
ATR35	_
ATR55	_
ATR63	NP_396031.1
CVR158	NP_902941.1
CVR22	NP_902547.1
PGR16	NP_905235.1
PGR18	NP_905312.1
PGR26	NP_906037.1
EFR167	_
EFR41	NP_816743.1
BER31	NP_881386.1
BER132	NP_881395.1
BER141	NP_881801.1

Table S3: List of primers used in this study

ORF ID	Primer 5'	Primer 3'
BA_3_15	GAGAACCTGTACTTTCAGGGTATGAAATACGCAAGATTAAC	GGGGACCACTTTGTACAAGAAAGCTGGGTTATTAACTAATGAGTTGATGTATG
BA_5_34	GAGAACCTGTACTTTCAGGGTATGAAAAAAAGAAATGATAATTTAAAAC	GGGGACCACTTTGTACAAGAAAGCTGGGTTATTATTAATCTATTTCGTCTTCAGC
BA_6_46	GAGAACCTGTACTTTCAGGGTATGGCTAGAGACGAAC	GGGGACCACTTTGTACAAGAAAGCTGGGTTATTATTTATATACTCAGCTTCC
BA_8_56	GAGAACCTGTACTTTCAGGGTATGATTTTCGATAAAGAAGAAAAC	GGGGACCACTTTGTACAAGAAAGCTGGGTTATTAGAAACCTAAATCGCG
BA_13_18	GAGAACCTGTACTTTCAGGGTATGTTGATAGATCATTTAGAATACAATTC	GGGGACCACTTTGTACAAGAAAGCTGGGTTATTAATACCTTTTTCTACTTCTATTT
BA_27_18	GAGAACCTGTACTTTCAGGGTATGAAAACACTCCGACTTATTTTAG	GGGGACCACTTTGTACAAGAAAGCTGGGTTATTAATAATTAAACGTTTTTAAATCCCAATTTC
BA_30_17	GAGAACCTGTACTTTCAGGGTATGTTTAAAGTAGGAGACG	GGGGACCACTTTGTACAAGAAAGCTGGGTTATTAATAAGTACTCACATTTTGG
BA_31_44	GAGAACCTGTACTTTCAGGGTATGTTTTCAGAAAAAGCCAATAAAATATTTC	GGGGACCACTTTGTACAAGAAAGCTGGGTTATTATTTTTGAGAACGCAATACTGG
BA_47_29	GAGAACCTGTACTTTCAGGGTATGGTGGAAAAGGAAATTGAAAGC	GGGGACCACTTTGTACAAGAAAGCTGGGTTATTAGCCATTATCTTTTGGTTTATATTTAC
BA_53_45	GAGAACCTGTACTTTCAGGGTATGAGTTTAGAAAAAGTATTATCAATAG	GGGGACCACTTTGTACAAGAAAGCTGGGTTATTATTCTTCCTCTTCAGTAGC
BA_212_10	GAGAACCTGTACTTTCAGGGTATGAATAAACAAAACTTTTTACAAAC	GGGGACCACTTTGTACAAGAAAGCTGGGTTATTATCCTATATATATAATGTATGCTAATG
BA_300_2	GAGAACCTGTACTTTCAGGGTATGAAAAACAACGGAAATAATAC	GGGGACCACTTTGTACAAGAAAGCTGGGTTATTACTATTTTAGAGTAAATTCTGATGTG
BA_2_7	GAGAACCTGTACTTTCAGGGTATGAAGCTATCAGAAGTTAAAAAAAAC	GGGGACCACTTTGTACAAGAAAGCTGGGTTATTAACAACAGCTACCGCC
BA_5_69	GAGAACCTGTACTTTCAGGGTATGTTGGAGGAAGAAGAATTTAAAG	GGGGACCACTTTGTACAAGAAAGCTGGGTTATTACTTCCTGCTGTTTAATTG
BA_6_27	GAGAACCTGTACTTTCAGGGTATGCTTTGGGCACATTATGC	GGGGACCACTTTGTACAAGAAAGCTGGGTTATTAATAAAATCCAGTAACAATTCTATG
BA_7_45	GAGAACCTGTACTTTCAGGGTATGAAAAATTTACTTGCTTTTTTACTGC	GGGGACCACTTTGTACAAGAAAGCTGGGTTATTATCACTCCGTTTTCTTAAAGTCC
BA_40_17	GAGAACCTGTACTTTCAGGGTATGGATAATACAATTATAATTGTAGGGG	GGGGACCACTTTGTACAAGAAAGCTGGGTTATTATTCTTCAGTTCCAATACGTTG
BA_43_2	GAGAACCTGTACTTTCAGGGTATGACGGGCCATGGG	GGGGACCACTTTGTACAAGAAAGCTGGGTTATTACTAGTTTTCAAAAACGTATTTTATAAT
BA_58_6	GAGAACCTGTACTTTCAGGGTATGAAAAAGCTTTACGTTATC	GGGGACCACTTTGTACAAGAAAGCTGGGTTATTACTAATCGACCTGAATAATAACG
BA_64_66	GAGAACCTGTACTTTCAGGGTATGACAACCGAGTTTTCTG	GGGGACCACTTTGTACAAGAAAGCTGGGTTATTATGCCATAGTAAGTTTAACAAC
BA_80_14	GAGAACCTGTACTTTCAGGGTATGTACGAAAAAACCTATCCAAATAAAC	GGGGACCACTTTGTACAAGAAAGCTGGGTTATTATAGTTTGATTCTCTCCGCATAAAC
BA_17	GAGAACCTGTACTTTCAGGGTATGAAACGATTAGACCCTAC	GGGGACCACTTTGTACAAGAAAGCTGGGTTATTACAGAATCTACAAAAGCACG
BA_119_21	GAGAACCTGTACTTTCAGGGTATGGCAGAACACAACG	GGGGACCACTTTGTACAAGAAAGCTGGGTTATTAAAAATGATAAAACGCATTCTC
BA_121_2	GAGAACCTGTACTTTCAGGGTATGGTGAAATTATTACATCGTATTGGG	GGGGACCACTTTGTACAAGAAAGCTGGGTTATTATTTCCAAATAACTTCAGTTATTGTAG
BA_42_3	GAGAACCTGTACTTTCAGGGTATGTCTAAAATAGAAGAGTTTTTAACAG	GGGGACCACTTTGTACAAGAAAGCTGGGTTATTATCCTTTTGAAATTGTGTTTGGTAATTC
BA_101_99	GAGAACCTGTACTTTCAGGGTATGGAAAATGAAATAATCAATAG	GGGGACCACTTTGTACAAGAAAGCTGGGTTACTATTTTCTTTTAAATAAAGGTAC
BA_75_21	GAGAACCTGTACTTTCAGGGTATGTTTCAATTAGGAAAAACCATAG	GGGGACCACTTTGTACAAGAAAGCTGGGTTACTATTTGAAATTCTTTTCAGCCAC
BA_150_2	GAGAACCTGTACTTTCAGGGTATGACCGTTATTGATCCGAATGC	GGGGACCACTTTGTACAAGAAAGCTGGGTTATTAAATAATTTTTTCACCATTTAAATTAG
BA_169_10	GAGAACCTGTACTTTCAGGGTATGAACACACAAAACCAAACCGTTG	GGGGACCACTTTGTACAAGAAAGCTGGGTTATTACAAGCTTGAAAAGTTAAGTG
BA_237_2	GAGAACCTGTACTTTCAGGGTATGCAATCTGAATCAGAAAAC	GGGGACCACTTTGTACAAGAAAGCTGGGTTATTACCACCAAACAATAAAG
